# Interleukin-10 Conjugation to Carboxylated PVP-Coated Silver Nanoparticles for Improved Stability and Therapeutic Efficacy

**DOI:** 10.3390/nano7070165

**Published:** 2017-07-02

**Authors:** Dieudonné R. Baganizi, Elijah Nyairo, Skyla A. Duncan, Shree R. Singh, Vida A. Dennis

**Affiliations:** Center for NanoBiotechnology Research, Alabama State University, 1627, Hall Street, Montgomery, AL 36101, USA; drbaganizi@gmail.com (D.R.B.); enyairo@alasu.edu (E.N.); skyyeduncan@gmail.com (S.A.D.); ssingh@alasu.edu (S.R.S.)

**Keywords:** interleukin-10, poly(vinylpyrolidone) (PVP), PVP-coated silver nanoparticles, bioconjugation, anti-inflammatory effect, storage stability, inflammatory mediators

## Abstract

Interleukin-10 (IL-10) is a key anti-inflammatory and immunosuppressive cytokine and therefore represents a potential therapeutic agent especially in inflammatory diseases. However, despite its proven therapeutic efficacy, its short half-life and proteolytic degradation in vivo combined with its low storage stability have limited its therapeutic use. Strategies have been developed to overcome most of these shortcomings, including in particular bioconjugation with stabilizing agents such as polyethylene glycol (PEG) and poly (vinylpyrolidone) (PVP), but so far these have had limited success. In this paper, we present an alternative method consisting of bioconjugating IL-10 to PVP-coated silver nanoparticles (Ag-PVPs) in order to achieve its storage stability by preventing denaturation and to improve its anti-inflammatory efficacy. Silver nanoparticles capped with a carboxylated PVP were produced and further covalently conjugated with IL-10 protein by carbodiimide crosslinker chemistry. The IL-10 conjugated Ag-PVPs exhibited increased stability and anti-inflammatory effectiveness in vitro. This study therefore provides a novel approach to bioconjugating PVP-coated silver nanoparticles with therapeutic proteins, which could be useful in drug delivery and anti-inflammatory therapies.

## 1. Introduction

Interleukin-10 (IL-10) is a major anti-inflammatory and immunosuppressive cytokine mainly produced by activated monocytes and macrophages, and different T-cell subsets [1,2]. IL-10 suppresses immune responses and inflammation by down-regulating the synthesis and expression of pro-inflammatory cytokines by macrophages and T cells including, Interleukin 1 beta (IL-1β), Interleukin 6 (IL-6), tumor necrosis factor (TNF) and Interferon gamma (IFN-γ), and by inhibiting the events related to antigen presentation [2,3]. IL-10 thus plays a major role in inflammatory diseases and autoimmune pathologies, and thereby represents a potential therapeutic agent for their treatment [1,4]. Recombinant IL-10 has, therefore, been targeted to treat inflammatory diseases such as psoriasis and inflammatory bowel diseases (IBD), however with very limited clinical progress [1,4,5]. Indeed, the clinical use of proteins, such as IL-10, is restricted by their stability, short half-life, and enzymatic degradation, thereby necessitating high dosages and frequent administration [6,7]. Furthermore, the handling and storage of proteins, especially freeze-thaw cycles and storage time, can affect their stability and contribute to their denaturation, which ultimately give rise to significant variations in their activity and efficacy [8,9]. Several approaches have been developed to counteract these problems, including, notably, conjugation with biocompatible and biodegradable polymers, and in particular, the conjugation with polyethylene glycol (PEG) [7]. PEGylated recombinant IL-10 has for instance provided some compelling results as an anti-tumor therapy [3,10].

Metal-based nanoparticles represent promising candidates as drug carriers. In fact, they have been proven to be excellent drug delivery vehicles, enabling their transport, bioavailability, and subsequent release to specific tissue sites [11]. Moreover, although hazards can be introduced by using metal nanoparticles as delivery systems, there are various approaches that can be employed to minimize this risk. Biocompatible polymers or capping agents such as poly(vinylpyrolidone) (PVP) and PEG are commonly used to reduce nanoparticle cytotoxicity, to improve their in vivo stability and to avoid their uptake by the reticular endothelial system [12,13]. PVP was found to exhibit the best stabilizing and protecting properties for nanoparticles [14,15]. Furthermore, these stabilizing/capping agents can be used as a site for bioconjugation of the nanoparticle with molecules of interest [12,16]. On the other hand, silver nanoparticles (Ag), in particular PVP-coated silver nanoparticles (Ag-PVPs), have been shown to possess potent anti-inflammatory properties [17,18,19]. Ag-PVPs were shown to reduce the levels of pro-inflammatory cytokines IL-6, TNF, IL-1α and IL-12p70 in *Chlamydia trachomatis*-infected macrophages and to down-regulate the mRNA gene transcript expressions of TLR2 and NOD2 induced by *C. trachomatis* [18]. In view of the above considerations, the ultimate goal of this study is to conjugate IL-10 to Ag-PVPs as an alternative for its therapeutic use. Our hypothesis is that IL-10 conjugated to Ag-PVPs will improve its stability and storage time by preventing denaturation and enhance its anti-inflammatory actions. First, we synthesized silver nanoparticles with a carboxylated PVP on the particle surface, thus enabling the post-conjugation with primary amines available on the IL-10 protein. Next, we showed the usefulness of IL-10 conjugation to Ag-PVPs in achieving increased IL-10 stability and improved anti-inflammatory efficacy. The data from this study provides evidence that PVP-coated silver nanoparticles can be exploited as drug delivery systems in anti-inflammatory therapies.

## 2. Results 

### 2.1. Activation of PVP with Carboxylic Acid Groups

**Figure 1:**
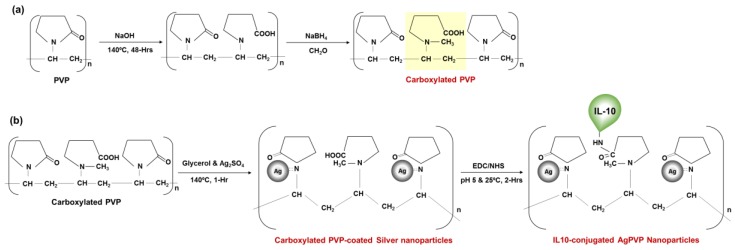
Representation of the production of Interleukin-10(IL-10) conjugated to carboxylated poly (vinylpyrolidone) (PVP)-coated silver nanoparticles. (**a**) Ring-opening and carboxylation of PVP by basic hydrolysis at high temperature and protection of the pyrrolidone nitrogen from ring closure; (**b**) Synthesis of Ag-PVP-COOH by the polyol method using silver sulfate as the precursor and glycerol as the reducing agent and solvent; and covalent conjugation of Ag-PVP-COOH with recombinant mouse IL-10 using EDC/NHS (1-ethyl-3-(3-dimethylaminopropyl) carbodiimide and N-Hydroxysuccinimide) chemistry.

**Figure 2:**
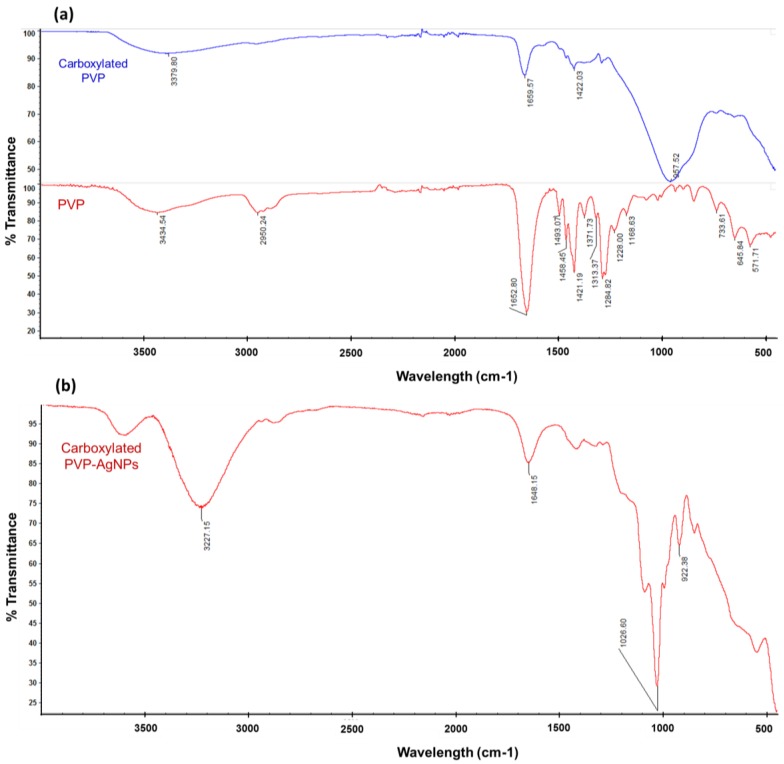
Fourier transform-infrared spectroscopy (FT-IR) spectra of (**a**) PVP and carboxylated PVP; and (**b**) dried carboxylated PVP-coated silver nanoparticles. The FT-IR spectra of carboxylated PVP show a change in the pyrrolidone C=O group peak at ~1659 cm^−1^ corresponding to the C=O stretching of the pyrrolidone ring confirming the ring opening; and a broad peak that centers at ~3379 cm^−1^ confirming the presence of carbonyl and hydroxyl moieties of the carboxylic acid group. On top: Carboxylated PVP; Bottom: PVP. The FTIR spectra of carboxylated Ag-PVPs show a strong band at ~1648 cm^−1^ of the carbonyl group stretching of PVP indicating the PVP capping of silver nanoparticles and a broad peak that centers at ~3227 cm^−1^ of the O–H and C–H stretching confirming the presence of carboxylic acid groups.

The PVP was carboxylated by opening and hydrolyzing the pyrrolidone ring by heating the PVP in a basic solution (NaOH) (Figure 1a), which was confirmed by Fourier transform-infrared spectroscopy (FT-IR) spectroscopy. The FT-IR spectra of PVP before activation and carboxylation (carboxylated PVP) are shown in Figure 2. The FT-IR spectrum of the non-activated PVP (Figure 2a; top graph) shows the characteristic stretching band of PVP located at ~1659 cm^−1^ corresponding to the pyrrolidone C=O group. Other important bands include those due to the C–N stretching vibrations and the –CH_2_ absorption of PVP at ~1284 cm^−1^, ~1421 cm^−^1, and ~1458 cm^−1^, and the absorption peak at ~1371 cm^−1^ due to the С–Н bond in PVP [20,21]. The FTIR absorption spectra of non-activated PVP also show clear absorption peaks located at ~2950 cm^−1^ and a large, broad peak that is centered at ~3434 cm^−1^, that are due to –OH symmetric stretching and –CH asymmetric stretching vibration peaks, respectively. The FT-IR spectrum of carboxylated PVP (Figure 2a; bottom graph) shows a change in the frequency of the characteristic band at ~1650 cm^−1^ of the pyrrolidone ring, confirming the ring opening. Furthermore, the spectrum of carboxylated PVP shows a large broad peak between 3000 and 3500 cm^−1^ that is centered at ~3379 cm^−1^ due to the O–H and C–H-stretching modes corresponding to the carboxylic acid groups. These observations indeed confirm that the PVP was successfully activated with the carboxylic acid groups.

Carboxylated PVP-coated silver nanoparticles were synthesized by the polyol method using silver sulfate and glycerine (Figure 1b). Prior to conjugation, the presence of the carboxylic acid groups on the synthesized nanoparticle surface was confirmed by FT-IR spectroscopy (Figure 2). Figure 2b shows the FT-IR peaks of dried carboxylated PVP-coated silver nanoparticles after spectral subtraction of the absorption of water. The peak observed at ~1648 cm^−1^ corresponds to the carbonyl group stretching of PVP and entails a coordinative bonding of C–N to Ag between the PVP and silver nanoparticles, and therefore confirms the PVP capping of the silver nanoparticles. Compared with the FT-IR spectrum of carboxylated PVP (Figure 2a; bottom graph), only a slight shift can be observed, suggesting that the oxygen atoms of the carboxylated PVP may not be involved in the bonding to the silver nanoparticles [20,21]. The peak at ~1028 cm^−1^ shows the presence of the –CN stretching vibration of PVP and could indicate the coordination between pyrrolidinyl nitrogen and Ag in the formation of the silver nanoparticles [20,21]. The peak at ~922 cm^−1^ can be attributed to fingerprints. It has been reported that the FT-IR spectrum of silver nanoparticles capped with PVP generally exhibits common features with the PVP, including bands at 2950 cm^−1^ and ~3400 cm^−1^ corresponding to –OH and –CH2 stretching vibration, respectively [20,21]. Conversely, the FT-IR spectrum of our nanoparticles show only a broad peak that centers at ~3227 cm^−1^ attributed to the O–H (and C–H) stretching of carboxylic acid groups. Therefore, these observations further proved that the carboxylic acid groups previously activated on the PVP were still available on the nanoparticle surface.

The amount of synthesized nanoparticles was calculated by UV-Vis quantification of PVP on the Ag-PVP NPs and was based on the reaction profile of the formation of silver nanoparticles. The PVP was recovered from the nanoparticles by centrifugation at 30,000 rpm for 30 min. The concentration of PVP was determined using a standard curve of PVP absorbance at 300 nm with PVP samples of a known concentration (diluted in Milli-Q water). The amount of the PVP obtained represents the yield reached during the synthesis of the nanoparticles. The concentration of Ag-PVP NPs could be directly related to the amount of PVP and calculated from the starting concentration of silver sulfate (0.015 M). In this way, the yield of the synthesis of the carboxylated PVP-coated silver nanoparticles was estimated to be >99%. The Ag-PVP NPs were re-suspended in sterile Milli-Q water at 1.3 mg/mL and stored at 4 °C.

### 2.2. IL-10 Conjugation to Carboxylated PVP-Coated Silver Nanoparticles

**Figure 3:**
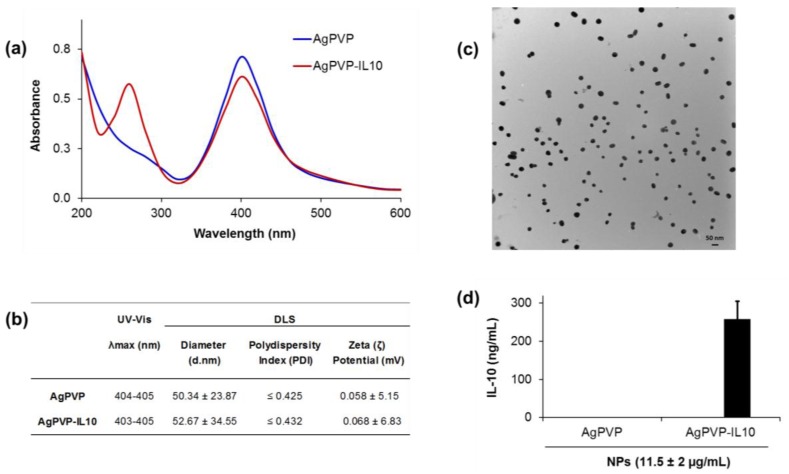
Characterization of IL10-conjugated Ag-PVPs. (**a**) UV-Vis spectra of rmIL-10 conjugated Ag-PVPs showing changes in absorption and an additional protein peak in the 280 nm region; (**b**) DLS characterization of Ag-PVPs before and after conjugation with rmIL10; (**c**) TEM image of dispersed rmIL-10 conjugated Ag-PVPs in aqueous solution (diameter size ~50 nm); (**d**) Enzyme-linked immunosorbent assay (ELISA) measurement of the amount of bioactive rmIL-10 conjugated to Ag-PVPs. The nanoparticle samples were analyzed immediately prior to use (after a week of storage). The data presented in (**a**) and (**b**) and are representative of one batch of nanoparticles, whereas the data presented in (**c**) and (**d**) are means and standard deviations of three independent samples run each in triplicate.

Carboxylated PVP-coated silver nanoparticles were later conjugated with rmIL-10 using (1-ethyl-3-(3-dimethylaminopropyl) carbodiimide and N-hydroxysuccinimide) chemistry (EDC/NHS) chemistry and characterized using UV-Visible spectroscopy to confirm the conjugation. The surface plasmon resonance (SPR) spectrum peaks of non-conjugated Ag-PVPs was found to be 404–405 nm and 403–405 nm after conjugation (Figure 3a,b), characteristic peaks of silver nanoparticles. Moreover, there is the appearance of an additional protein peak in the 280 nm region (Figure 3a), thereby confirming the successful covalent conjugation of rmIL-10 to the nanoparticles. The hydrodynamic size and surface-charge zeta potential measurements performed with a Malvern Zetasizer Nano ZS in neutral water (pH 7) showed a smaller shift or no shift at all. The zeta potential of Ag-PVPs was 0.058 (±5.15) mV and 0.068 (±6.83) mV for the rmIL-10 conjugated Ag-PVPs (Figure 3b). The hydrodynamic diameter of the non-conjugated Ag-PVPs was 50.34 nm, whereas it was 52.67 nm after conjugation with Recombinant Mouse IL-10 (rmIL10) (Figure 3b). As shown in Figure 3c; the TEM analysis revealed that the rmIL-10 conjugated Ag-PVPs were roughly spherical in shape with a diameter size of the Ag-core averaging 50 (±10) nm as estimated from the TEM images by the Image J software (ImageJ 1.48v, Wayne Rasband, National Institutes of Health, Bethesda, MD, USA) (Figure 3c). The amount of bioactive rmIL-10 on the Ag-PVPs averaged a ratio of 1 ng/mL rmIL-10 for 45 ng/mL of Ag-PVPs, as measured by enzyme-linked immunosorbent assay (ELISA) (Figure 3d).

### 2.3. Cytotoxicity Effect of rmIL-10 Conjugated Ag-PVPs

**Figure 4:**
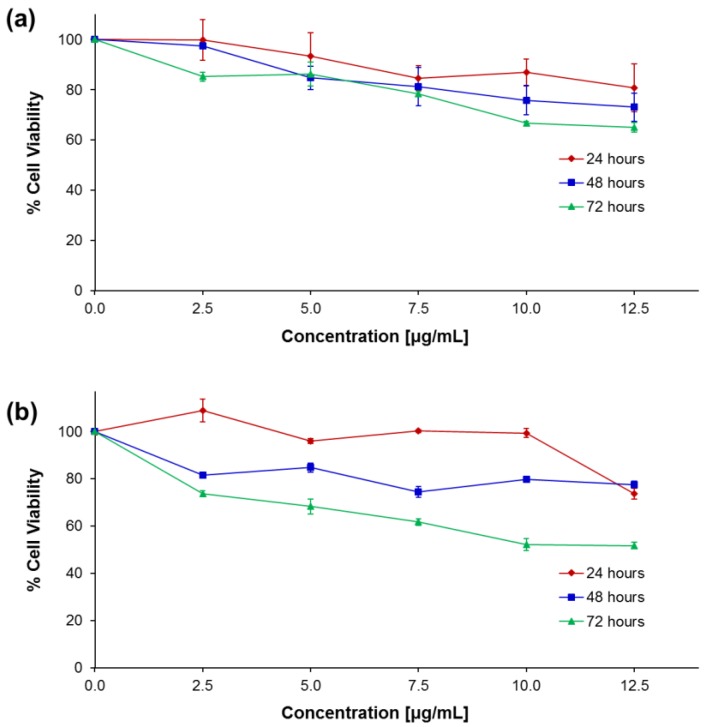
The effect of rmIL-10-conjugated Ag-PVPs (**a**) and non-conjugated Ag-PVPs (**b**) on cellular viability. Mouse J774 macrophages were exposed to Ag-PVPs or rmIL-10-conjugated Ag-PVPs for 24, 48, and 72 h and the cell viability was determined by the tetrazolium dye MTT (3-(4,5-dimethylthiazol-2-yl)-2,5-diphenyltetrazolium bromide) based assay. The relative survival of cells is expressed as the percent of control untreated cells.

The potential cytotoxic effect of rmIL-10 conjugated Ag-PVPs and non-conjugated Ag-PVPs to mouse J774 macrophages was evaluated by tetrazolium dye MTT (3-(4,5-dimethylthiazol-2-yl)-2,5-diphenyltetrazolium bromide) based assay for cell viability to ensure that the anti-inflammatory effects of conjugated rmIL-10 are not related to nanoparticle-induced cell death. After up to 72 h exposure, the concentrations of rmIL-10 conjugated Ag-PVPs (at <5 μg/mL) showed very good cell viability (≥85% viable cells) (Figure 4a). The concentrations above 7.5 μg/mL showed noticeable cell death with ≥30% decline in cell viability after 48 and 72 h (Figure 4a). On the other hand, non-conjugated Ag-PVPs gave similar results at 24 and 48 h of incubation, but showed higher toxicity after 72 h with ≤30% decline in cell viability at only <2.5 μg/mL (Figure 4b). The concentrations of rmIL-10 conjugated Ag-PVPs used in this study were below 1 μg/mL; a concentration range resulting in less than 15% decline in cell viability after up to 72 h. Overall, the MTT results did not reveal any severe cytotoxicity of the rmIL-10 conjugated Ag-PVPs to mouse macrophages, especially at lower concentrations.

### 2.4. rmIL-10 Conjugated Ag-PVPs Reduced LPS-Induced Inflammatory Responses

**Figure 5:**
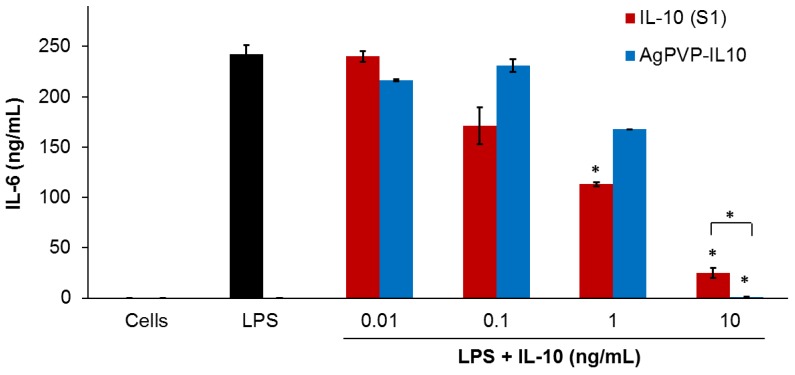
The effect of free rmIL-10 and rmIL-10 conjugated Ag-PVPs on the production of IL-6 by mouse J774 macrophages stimulated with lipopolysaccharide (LPS). Cells (10^6^ cells/mL) were stimulated with LPS (1 μg/mL) and treated with different concentrations of either free rmIL-10 (fresh, S1) or rmIL10-conjugated Ag-PVPs (stored one week at 4 °C). Cell-free culture supernatants were collected at 72 h post-incubation and were used to measure the production of IL-6 by cytokine ELISA. Untreated and LPS-stimulated cells were used as positive controls, and untreated and non-stimulated cells were used as negative controls. * indicates a significant difference (*p* < 0.01) between the untreated and LPS-stimulated cells versus LPS-stimulated and treated cells as calculated by the unpaired Student’s *t*-test. The data are presented as means and standard deviations of samples run in duplicate and are representative of three separate experiments.

**Figure 6:**
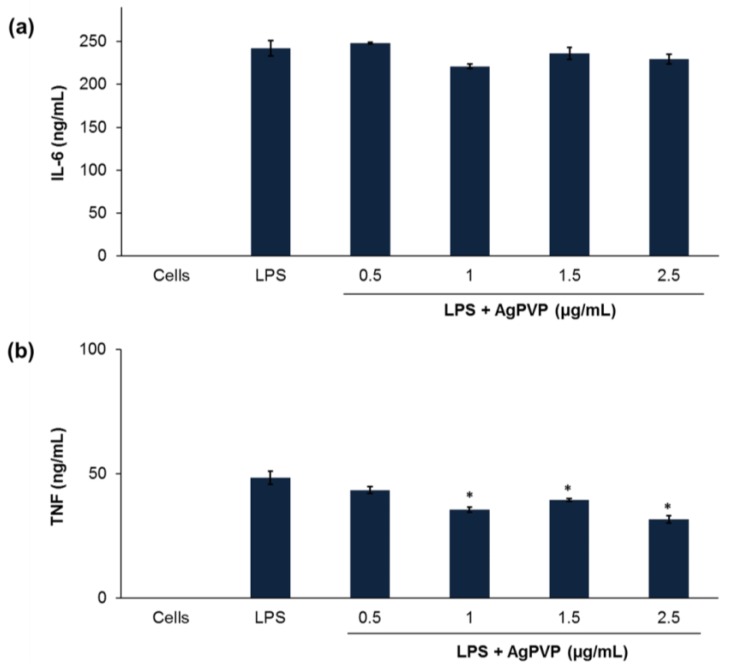
The effect of non-conjugated Ag-PVPs on the production of IL-6 (**a**) and tumor necrosis factor (TNF) (**b**) by mouse J774 macrophages stimulated with LPS after 72 h. Cells (10^6^ cells/mL) were stimulated with LPS (1 μg/mL) and treated with different concentrations of Ag-PVPs (stored one week at 4 °C). Cell-free culture supernatants were collected at 72 h post-incubation and used to measure the production of IL-6 by cytokine ELISA. Untreated and LPS-stimulated cells were used as positive controls, and untreated and non-stimulated cells were used as negative controls. * indicates a significant difference (*p* < 0.01) between the untreated and LPS-stimulated cells versus LPS-stimulated and treated cells as calculated by the unpaired Student’s *t*-test. The data are presented as means and standard deviations of samples run in triplicate and are representative of three separate experiments.

The stimulation of mouse J774 macrophages by LPS results in the activation of a series of signaling events that led to the secretion of several inflammatory mediators [22]. IL-6, TNF, and IL-1β represent the most important mediators of inflammatory responses and are therefore the preferred inflammatory markers [23]. Hence, we investigated the anti-inflammatory effects of rmIL-10 conjugated Ag-PVPs (average ratio of 1:45 of bioactive rmIL-10 on Ag-PVPs) on LPS-induced IL-6 and TNF production in mouse macrophages. Three different samples of free rmIL-10 were used: fresh rmIL-10, rmIL-10 after one freeze-thaw cycle, and rmIL-10 stored for one week at 4 °C. These were the same conditions of storage as that for rmIL-10 conjugated Ag-PVPs and non-conjugated Ag-PVPs. Mouse J774 macrophages stimulated with LPS secreted high levels of IL-6 when compared to the levels from the unstimulated cells (Figure 5). When LPS-stimulated macrophages were exposed to 0.01, 0.1, 1, and 10 ng/mL of fresh rmIL-10, they secreted less IL-6 in a dose-dependent fashion, with the 1 and 10 ng/mL significantly decreasing the IL-6 secretion (Figure 5). Treatment of LPS-stimulated macrophages with fresh rmIL-10 conjugated Ag-PVPs also resulted in a dose-dependent reduction of IL-6 secretion, but with only a marked decrease at 10 ng/mL (Figure 5). These effects were observed at 72 h post-incubation for both fresh the rmIL-10 and fresh rmIL-10 conjugated to Ag-PVPs without changing the culture medium (Figure 5). A short time incubation (24 and 48 h) also resulted in similar effects (data not shown). On the other hand, the treatment of LPS-stimulated macrophages with non-conjugated Ag-PVPs at concentrations ranging from 0.5 to 2.5 μg/mL resulted in a slight and non-significant reduction of IL-6 secretion (Figure 6a). It is worth noting that although fresh rmIL-10 conjugated to Ag-PVPs was less efficient at low concentrations compared to the fresh rmIL-10, when used at a high concentration (10 ng/mL), it was significantly more effective than fresh rmIL-10 at reducing the IL-6 levels (*p* < 0.01).

**Figure 7:**
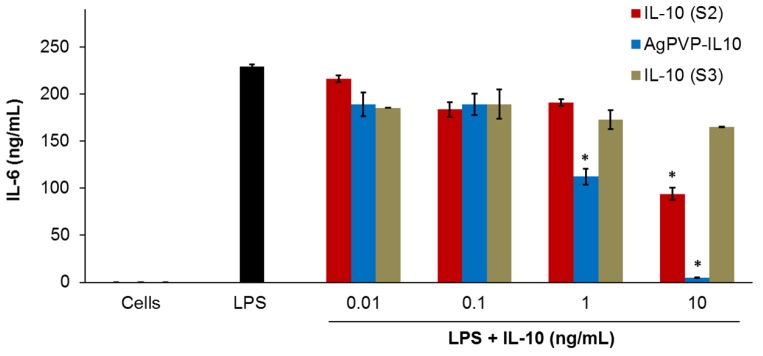
The effect of storage of free rmIL-10 and rmIL-10 conjugated to Ag-PVPs on the production of IL-6 by mouse J774 macrophages stimulated with LPS. Cells (10^6^ cells/mL) were stimulated with LPS (1 μg/mL) and treated with different concentrations of either free rmIL-10 (after one freeze-thaw cycle, S2), free rmIL-10 (stored one week at 4 °C, S3), or rmIL10-conjugated Ag-PVPs (stored one week at 4 °C). Cell-free culture supernatants were collected at 72 h post-incubation and were used to measure the production of IL-6 by cytokine ELISA. Untreated and LPS-stimulated cells were used as positive controls, and untreated and non-stimulated cells were used as negative controls. * indicates a significant difference (*p* < 0.01) between the untreated and LPS-stimulated cells versus LPS-stimulated and treated cells as calculated by the unpaired Student’s *t*-test. The data are presented as means and standard deviations of samples run in duplicate and are representative of two separate experiments.

On the other hand, the rmIL-10 after one freeze-thaw cycle resulted in a small reduction of the level of secreted IL-6; whereas there was no reduction of the IL-6 levels observed with rmIL-10 stored one week at 4 °C (Figure 7). Indeed, the decrease in IL-6 secretion by LPS-stimulated macrophages following a treatment with rmIL-10 after one freeze-thaw cycle and 72 h incubation was only statistically significant with 10 ng/mL of rmIL-10 (*p* < 0.01) (Figure 7). In contrast, rmIL10-conjugated Ag-PVPs stored one week at 4 °C led to a significant decrease of the IL-6 secretion at 1 and 10 ng/mL (Figure 7), as efficiently as fresh rmIL-10 conjugated Ag-PVPs. Furthermore, there were no differences between short and long post-treatment incubation periods; indeed, the above observations remained unchanged after 24 and 48 h of incubation for all the compounds (data not shown). These observations therefore indicate that the handling and storage of rmIL-10 greatly affect its anti-inflammatory effect efficacy, which does not occur with rmIL-10 conjugated Ag-PVPs.

**Figure 8:**
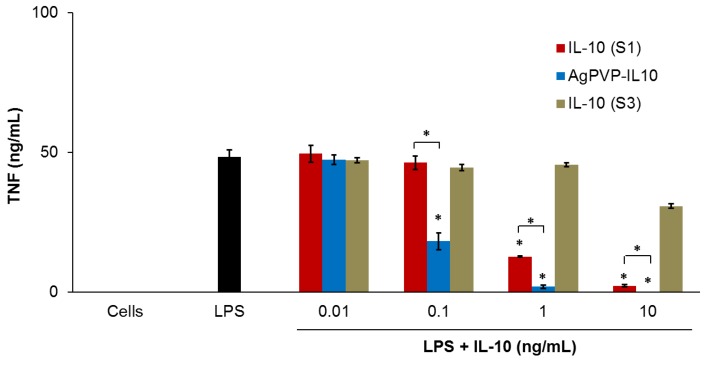
The effect of free rmIL-10 (fresh, S1), rmIL-10 conjugated to Ag-PVPs (stored one week at 4 °C), and free rmIL-10 stored one week at 4 °C (S3) on the production of TNF by mouse J774 macrophages stimulated with LPS. Cells (10^6^ cells/mL) were stimulated with 1 μg/mL LPS and treated with different concentrations of either free rmIL-10 or rmIL10-conjugated Ag-PVPs. Cell-free culture supernatants were collected at 72 h post-incubation and titrated by ELISA. Untreated and LPS-stimulated cells were used as positive controls, and untreated and non-stimulated cells were used as negative controls. * indicates a significant difference (*p* < 0.01) between the untreated and LPS-stimulated cells versus LPS-stimulated and treated cells as calculated by the unpaired Student’s *t*-test. The data are presented as means and standard deviations of samples run in triplicate and are representative of three separate experiments.

These data were further confirmed by measuring the levels of TNF secreted by mouse J774 macrophages stimulated with LPS. LPS-stimulated cells also produced a high amount of TNF when compared to unstimulated cells (Figure 8). Both fresh rmIL-10 and rmIL-10 conjugated Ag-PVPs stored one week at 4 °C resulted in a dose-dependent reduction of TNF secretion, with 1 ng/mL and 10 ng/mL substantially decreasing the TNF secretion (*p* < 0.01) after 72 h of incubation (Figure 8). Ag-PVPs at concentrations ≥1 μg/mL also resulted in a statistically significant reduction of TNF secretion in a dose dependent manner, although to a lesser degree (Figure 6). Short time incubation (24 and 48 h) also resulted in similar results for all the compounds (data not shown). In addition, fresh rmIL10-conjugated Ag-PVPs achieved the same results as the rmIL10-conjugated Ag-PVPs stored one week at 4 °C (data not shown). Similar for IL-6, there was very little or no reduction of TNF levels observed with rmIL-10 stored one week at 4 °C (Figure 8). Interestingly, rmIL-10 conjugated Ag-PVPs were more effective at reducing TNF levels than fresh rmIL-10, especially at 1 ng/mL and 10 ng/mL (*p* < 0.01) (Figure 8), which resulted in very little or no detection of TNF, which suggests an additive effect provided by the anti-inflammatory activity of Ag-PVPs on the reduction of TNF (Figure 6). Since the concentrations of Ag-PVPs were not toxic to cells (Figure 4), this eliminates the possibility that the anti-inflammatory effects of rmIL-10 conjugated Ag-PVPs could be due to nanoparticle-induced cell death. These findings demonstrate that the conjugation of IL-10 to Ag-PVPs allows for protecting IL-10 against denaturation and improving its anti-inflammatory efficacy.

## 3. Discussion

IL-10 is a cytokine with potent anti-inflammatory and immunoregulation properties that have now been well-established in inflammatory diseases, various infections, and in antitumor immunity [1,3,5]. This has generated a high interest in the use of recombinant IL-10 as an anti-inflammatory therapy and anticancer immunotherapy; however the limited clinical progress observed has considerably narrowed the therapeutic application of IL-10 [1]. The therapeutic use of IL-10, like other bioactive proteins, suffers from the lack of stability and proteolytic degradation in vivo, access to targets, and dose-dependent side effects [6,7]. Moreover, the time and temperature of storage and the handling of bioactive proteins must also be considered, as they affect their stability and/or contribute to their denaturation, which leads to fluctuations in their activity and/or reduced effects [8,9,24]. Chemical bioconjugation with water-soluble and biocompatible polymers allows for overcoming most of these problems. They allow, in particular, to minimize the proteolytic cleavage, to increase the biodistribution, and to decrease the side effects, which result in improved therapeutic effects [7,25]. Therefore, in the present study, we performed a bioconjugation of IL-10 to PVP-coated silver nanoparticles, which also have anti-inflammatory properties [17,18,19], in order to improve its storage stability and anti-inflammatory efficacy as an alternative for its therapeutic use. For this purpose, we have established a methodology with sequential strategies allowing us to obtain monodisperse shape- and size-controlled silver nanoparticles capped with carboxylic acid-bearing PVP enabling further conjugation with IL-10. We further demonstrated the improved stability and anti-inflammatory effect of the bioconjugated IL-10 in mouse macrophages.

Among the polymers used for bioconjugation, PVP has been found to be the most advantageous. Indeed, PVP has been shown to have the longest circulation time compared with other common polymers such as PEG and dextran [25], and to enable restricted localization, longer plasma half-life, and more potent activity of the conjugated bioactive proteins in tissues without increasing their side effects [7,25]. Moreover, PVP is an excellent stabilizing and protecting agent for metal nanoparticles, enabling the control of the size distribution, the formation of specific shapes, and the aggregation process of the particles [14,15]. However, unlike other polymers such as PEG, PVP is a homopolymer, composed of a polyvinyl backbone with individual repeating units containing a polar amide group and non-polar methylene groups both in the backbone and in the ring [15,20]. The N and O atoms of the PVP polar group have a strong affinity for the silver ions and silver nanoparticles, and thereby coordinate to form a covered layer on the surface of the nanoparticles during their synthesis [15,20]. Hence, PVP capped silver nanoparticles lack the ability to further conjugate biomolecules to the nanoparticle surface. Consequently, in this study, the PVP was activated with carboxylic acid (–COOH) groups prior to the synthesis of nanoparticles.

Several approaches are now available and efficient for introducing useful functional groups onto PVP. The radical copolymerization of 1-Vinyl-2-pyrrolidinone (VP) with an organic solvent as a radical initiator (e.g., azobisisobutyronitrile or 4,4′-Azobis-(4-cyanovaleric acid), ACVA) and a chain transfer agent (e.g., mercaptoacetic acid or mercaptpropionic acid) is one of the most efficient and effective ways to produce terminal carboxyl-bearing PVP [26,27,28]. This method has for instance allowed for further covalent conjugation of bioactive TNF and IL-6 [27,28], but was ineffective in our study as the synthesized carboxyl-terminated PVP did not allow the formation of silver nanoparticles. The intramolecular catalysis in the previously opened pyrrolidone ring of PVP was also shown to be suitable for covalent conjugation of primary and secondary functional amines of proteins with PVP [29,30]. This approach involves the pyrrolidone ring opening and hydrolysis with strong bases (e.g., KOH, NaOH) at high temperature to produce a carboxylic acid, followed by protection of the pyrrolidone nitrogen from ring closure during further conjugation [29,30]. We have therefore adapted the method from von Specht et al. [30], which in their study, allowed for obtaining a relatively high amount of ring-opening. Moreover, it has been demonstrated with polystyrene particles that the particles synthesized with such activated PVP could enable successful covalent bioconjugation to their surface [31]. However, since the oxygen and nitrogen atoms of the PVP ring are involved in the adsorption of PVP chains onto the nanoparticle surface [15,20], opening the PVP rings could thus affect the synthesis of nanoparticles.

Based on previous reports, which revealed in silver nanoparticles with a diameter size below 50 nm that only the nitrogen atom in the PVP ring was involved in the coordinative bonding onto silver nanoparticles [32], we aimed to synthesize small nanoparticles to limit this potential issue. Furthermore, small-sized PVP-coated silver nanoparticles were of high interest in this study as it was reported that their cytotoxicity decreases with their size and small-sized PVP-coated silver nanoparticles exhibit greater anti-inflammatory effects [18]. Silver nanoparticles were thus synthesized following the polyol synthesis method, which involves the reduction of silver precursor and dissolution of a stabilizing/protecting agent by a polyol [33,34]. This method is known to produce highly monodispersed and small-sized spherical silver nanoparticles [33,34]. With this method, the carboxylic acid-activated PVP successfully formed a protection layer on silver nanoparticles through the N atoms in the carboxylated PVP and the carboxylic acid groups remained available on the particle surface. Ag-PVP nanoparticles further allowed a successful covalent conjugation with rmIL-10 after the activation of the carboxylic acid groups by EDC/NHS. The rmIL-10 conjugated Ag-PVPs obtained were spherical in shape with a diameter size averaging 50 (±10) nm. However, it should be noted that the attachment of a relatively high number of polymer molecules to amine residues within a protein might increase the binding site disruption or reduce the affinity, which therefore affects its biological activity [35,36]. Moreover, unlike other cytokines, it has been noted that the covalent bioconjugation of IL-10 by chemical modification of lysine residues in IL-10 might disrupt the dimeric structure of IL-10 and potentially generate conjugated monomers with no biological activity [37]. Reportedly, the PEGylation of IL-10 on amino acid residues of IL-10 substantially reduced its in vitro biological activity [36]. However, in our study the best anti-inflammatory effect displayed by rmIL-10 conjugated Ag-PVPs at 10 ng/mL and its good binding to the capture antibody in ELISA suggest that the above effects did not occur during the conjugation of rmIL-10 to Ag-PVPs or that the amount of rmIL-10 monomer conjugates were negligible.

Despite the advantages offered by surface modified silver nanoparticles as drug delivery systems, such as bioavailability, release to specific sites, and modulation of side effects of the drug [38,39], their potential cytotoxicity is a matter for consideration. Studies have thus reported that capped silver nanoparticles induced toxic effects mainly related to their size and surface modification, which influence their aggregation in vitro and in vivo [40,41]. Notwithstanding, PVP capped silver nanoparticles cause negligible toxic effects as they maintain a good stability that prevents them from agglomeration [41]. In our study, over 85% of cells remained viable after up to 72 h exposure to rmIL-10 conjugated Ag-PVPs at nanoparticle concentrations reaching 5 μg/mL, indicating that these concentrations are non-toxic to cells. Moreover, the concentrations of silver nanoparticles used in this study were low enough to circumvent inducing any toxicity to cells (<1 μg/mL). The rmIL-10 conjugated Ag-PVPs strongly inhibited IL-6 and TNF in mouse macrophages stimulated with LPS in a concentration-dependent manner. Moreover, rmIL-10 conjugated to Ag-PVPs showed a more potent anti-inflammatory effect at 10 ng/mL than native rmIL-10. However, at low concentrations of rmIL-10 (0.1 and 1 ng/ mL), rmIL-10 conjugated Ag-PVPs were less effective than native rmIL-10 at reducing IL-6 levels while they were significantly more efficient at reducing TNF levels than fresh rmIL-10. This observation could be due to a variety of factors, including the mechanisms by which IL-10 exerts its inhibitory effects on these cytokines. Studies have thus shown that IL-10 has significant inhibition of LPS-induced TNF production while its inhibitory effects are less pronounced for IL-6 [42,43]. Moreover, although the non-conjugated AgPVPs exhibited very low anti-inflammatory effects at lower concentrations (i.e., <0.5 μg/mL), they led to a significant decrease of TNF secretion, which could have a beneficial effect on the activity of rmIL-10 conjugated Ag-PVPs at reducing TNF levels. However, with an average ratio of 1:45 of bioactive rmIL-10 on Ag-PVPs obtained after conjugation, the concentrations of silver nanoparticles used in our study were lower than those that have significant anti-inflammatory effects [18], hence limiting the possible additive effect of Ag-PVPs at low concentrations of IL-10.

On the other hand, in addition to improving the anti-inflammatory effect, rmIL-10 stability was also enhanced by its conjugation to Ag-PVPs. The stability of proteins in vitro is a major limiting factor for their therapeutic application. Indeed, unsuitable conditions of storage or transport often leads to their degradation and/or inactivity, and freeze-thaw cycles decrease their stability [8,9,24]. The bioconjugation of proteins with polymers has been demonstrated to improve their in vitro stability by protecting them from thermal inactivation, increasing the thermodynamic stability, and preventing the preservative-induced aggregation in solution [44,45,46]. In our study, free rmIL-10 also exhibited decreased activity or no activity following a freeze-thaw cycle or storage at 4 °C (Figure 7 and Figure 8). Conversely, rmIL-10 conjugated Ag-PVPs did not necessitate special measures with regard to storage conditions as they were stored at 4 °C and did not show variation in terms of effectiveness. Moreover, rmIL-10 conjugated Ag-PVPs have maintained their level of activity after up to three weeks at 4 °C (data not shown).

## 4. Materials and Methods 

### 4.1. Carboxylation of PVP

PVP was carboxylated by partially hydrolyzing the pyrrolidone ring following a previously published method that allows an amount of ring opening of 15% [30] (Figure 1a). Briefly, 0.2 g of PVP (MW = 29 kDa, Sigma Aldrich, St. Louis, MO, USA) was dissolved in 10 mL of 0.1 N NaOH and heated at 140 °C for 48 h in a beam calorimeter (Parr Instrument Company, Moline, IL, USA). In order to prevent the closing of the opened pyrrolidone ring, its γ-amino butyric was methylated by adding 600 μL of 35% formaldehyde solution (Sigma Aldrich, St. Louis, MO, USA) followed by adjusting the solution to pH 9 and then cooling to 0 °C. Next, 1.5% of sodium tetrahydroborate (Sigma Aldrich, St. Louis, MO, USA) was added; the solution was stirred for 45 min at room temperature (RT) and then vacuum-dried at 60 °C overnight. Prior to use, the carboxylation of PVP was assessed by Fourier transform-infrared spectroscopy (FT-IR) to monitor the ring opening and the presence of the carboxyl groups. FT-IR spectra were acquired with an FT-IR Nicolet 6700 (ThermoFisher Scientific Inc., Waltham, MA, USA) equipped with an attenuated total reflectance (ATR) stage, with 64 scans per sample with a resolution of 4 cm^−1^.

### 4.2. Synthesis of Carboxylated PVP-Coated Silver Nanoparticles

Carboxylated PVP-coated silver nanoparticles were synthesized by the polyol method using glycerin as a reducing agent and solvent as previously reported [47] (Figure 1b). Briefly, carboxylated PVP (0.2 g) was dissolved in 30 mL of glycerin and heated at 140 °C. After 30 min of heating, 2 mL of 0.015 M silver sulfate (Ag_2_SO_4_, Sigma Aldrich, St. Louis, MO, USA) was added and left to react for 1 h. Silver nanoparticles were then cooled at RT and the glycerin was removed by repeated (2 or 3 times) addition of ethanol and centrifugation (10,000 rpm) for 10 min at RT. The supernatant was removed and carboxylated PVP-coated silver nanoparticles were suspended in sterile distilled water and cooled at 4 °C in the dark. Prior to use, an aliquot of the nanoparticles was vacuum-dried and the presence of carboxyl groups on the nanoparticle surface was assessed by FT-IR spectroscopy as described above.

### 4.3. IL-10 Conjugation and Characterization of Nanoparticles

Recombinant mouse IL-10 (BioLegend, San Diego, CA, USA) was covalently conjugated to carboxylated PVP-coated silver nanoparticles using 1-ethyl-3-(3-dimethylaminopropyl) carbodiimide (EDC, Sigma Aldrich) and *N*-Hydroxysuccinimide (NHS, Sigma Aldrich) conjugation method (Figure 1b). Briefly described, 50 μL of carboxylated PVP-coated silver nanoparticles (1 mg/mL) was mixed with 50 μL of a solution of EDC/NHS (30/36 mg/mL) in 2-(*N*-morpholino) ethanesulfonic acid (MES) buffer (10 mM, pH 5.5) along with 25 μL of rmIL-10 (0.2 mg/mL) and vortexed for 2 h at RT. This concentration of rmIL-10 was used to allow saturation of the free carboxyl groups on the carboxylated PVP-coated silver nanoparticles. Unbound IL-10 was removed by washing with PBS and 0.05% Tween 20 (PBS/T) followed by centrifugation at 12,000 rpm for 30 min to obtain rmIL-10 conjugated to carboxylated PVP-coated silver nanoparticles (rmIL-10 conjugated Ag-PVPs). The conjugated IL-10 was re-suspended in sterile PBS at 1 mg/mL. An aliquot of the conjugated nanoparticles was diluted to 100 μg/mL in sterile distilled water and characterized using UV-Vis spectrophotometry and a dynamic light scattering system. UV-Vis analysis was used to monitor the absorption spectra and surface plasmon bands with a 1 cm path length using a NanoDrop 2000c spectrophotometer (ThermoFisher Scientific Inc., Waltham, MA, USA). The hydrodynamic size and zeta potential measurements of rmIL-10 conjugated Ag-PVPs were assessed using Malvern Zetasizer Nano ZS (Malvern Instruments Ltd., Westborough, MA, USA).

The size and shape of rmIL-10 conjugated Ag-PVPs were monitored by Transmission electron microscopy (TEM) analysis. The nanoparticles were mounted on carbon-coated formvar grids (Electron Microscopy Sciences Formvar/C Film SQ Grid 300 CU, Fisher Scientific, Pittsburgh, PA, USA), pre-exposed to 1% Alcian blue (Sigma Aldrich), air dried at RT and then observed under a Zeiss EM10 TEM Microscope (Carl Zeiss, Inc., Meditec, Oberkochen, Germany) operating at 60 kV. The amount of bioactive rmIL-10 on Ag-PVPs was determined by Enzyme-linked immunosorbent assay (ELISA; OptEIA™ Mouse IL-10 ELISA Set, BD Biosciences, San Jose, CA, USA) according to the manufacturer’s instructions.

### 4.4. Cell Culture

Mouse J774 macrophages (J774A.1; ATCC^®^ TIB-67™) were obtained from the American Type Culture Collection (ATCC, Manassas, VA, USA) and cultured based on a previously described protocol [48]. Briefly, cells were cultured at 37 °C in a humidified 5% CO_2_ atmosphere in Dulbecco’s Modified Eagle Medium (DMEM, GIBCO^®^, Life Technologies, Grand Island, NY, USA) supplemented with 10% heat-inactivated fetal bovine serum (FBS), 2 mM L-glutamine, and 1 μg/mL antibiotic and antimycotic.

### 4.5. Cytotoxicity Study

The cytotoxicity of rmIL-10 conjugated Ag-PVPs and non-conjugated Ag-PVPs to mouse J774 macrophages was evaluated by the 3-(4,5-dimethyl-thiazol-2-yl)-2,5-diphenyl-tetrazoliumbromide (MTT) dye reduction assay using a Cell-Titer 96^®^ Non-Radioactive Cell Proliferation Assay kit (Promega, Madison, WI, USA) as previously described [18]. Cells (10^5^ cells/well) were seeded in a 96-well plate in 100 μL of culture media and incubated overnight at 37 °C in a humidified 5% CO_2_ atmosphere. The culture media was replaced by 100 μL of fresh media containing various concentrations of rmIL-10 conjugated Ag-PVPs (2.5 to 12.5 μg/mL) for 24, 48, and 72 h. After incubation, the MTT dye solution (15 μL) was added to each well, and further incubated for 2 h. The reaction was stopped by adding 100 μL of solubilization solution/stop mixture to each well. The absorbance was measured at 570 nm using a microplate reader (Tecan™ Instruments, San Jose, CA, USA) and the cell viability was determined by comparing the ratio of absorbance of control cells incubated with the culture medium only ([A]_control_) to that of cells incubated with nanoparticles ([A]_test_) as follows: percent viability = [A]_test_/[A]_control_ × 100.

### 4.6. Cell Activation and Analysis of IL-10 Activity

Mouse J774 macrophages were stimulated with 1 μg/mL of Lipopolysaccharide (LPS from *Escherichia coli*, ThermoFisher Scientific) and were treated with different concentrations of either free rmIL-10, rmIL10-conjugated Ag-PVPs (0.01, 0.1, 1, and 10 ng/mL), or non-conjugated Ag-PVPs (0.5 to 2.5 μg/mL) and incubated at 37 °C in a humidified 5% CO_2_ atmosphere. Untreated and LPS-stimulated cells were used as positive controls, and untreated and non-stimulated cells were used as negative controls. Cell-free supernatants were collected after 72 h of incubation and the levels of secreted pro-inflammatory cytokines, IL-6, and TNF were measured by cytokine specific ELISAs using BD OptEIA™ sets for mouse IL-6 or TNF (BD Biosciences, San Jose, CA, USA), according to the manufacturer’s instructions.

### 4.7. Statistical Analysis

All experiments were repeated at least three times, and the results are presented as means and standard deviations. The unpaired Student’s *t*-test was used to compare differences between samples. The differences were considered statistically significant if *p* < 0.01 (*).

## 5. Conclusions

In summary, silver nanoparticles capped with a carboxylated PVP were successfully produced to achieve covalent bioconjugation with IL-10 protein. The IL-10 conjugated to Ag-PVPs exhibited enhanced stability and therapeutic efficacy in vitro. To our knowledge, this is the first attempt that demonstrates a successful conjugation of a bioactive protein to PVP-coated silver nanoparticles as well as providing evidence that PVP-coated silver nanoparticles could be explored as a drug delivery or targeting system.
